# Prospective association of daily toothbrushing frequency and the prevalence of childhood functional constipation: the Japan Environment and Children’s Study

**DOI:** 10.1038/s41598-025-88562-8

**Published:** 2025-03-05

**Authors:** Masahiro Tsuchiya, Shinobu Tsuchiya, Haruki Momma, Ryoichi Nagatomi, Nobuo Yaegashi, Takahiro Arima, Chiharu Ota, Kaoru Igarashi, Yuichiro Miura, the Japan Environment and Children’s Study Group, Nobuo Yaegashi, Michihiro Kamijima, Shin Yamazaki, Yukihiro Ohya, Reiko Kishi, Koichi Hashimoto, Chisato Mori, Shuichi Ito, Zentaro Yamagata, Hidekuni Inadera, Takeo Nakayama, Tomotaka Sobue, Masayuki Shima, Seiji Kageyama, Narufumi Suganuma, Shoichi Ohga, Takahiko Katoh

**Affiliations:** 1 Department of Nursing, Tohoku Fukushi University, 6-149-1 Kunimi-ga-oka, Aoba-ku, Sendai, Miyagi 981-3201 Japan; 2 Division of Craniofacial Anomalies, Tohoku University Graduate School of Dentistry, Sendai, 980-8575 Japan; 3 Department of Orthodontics and Speech Therapy for Craniofacial Anomalies, Tohoku University Hospital, Sendai, 980-8574 Japan; 4 Department of Medicine and Science in Sports and Exercise, Tohoku University Graduate School of Medicine, Sendai, 980-8575 Japan; 5 Designing Future Health Initiative, Promotion Office of Strategic Innovation, Tohoku University, Sendai, 980-8577 Japan; 6 Department of Obstetrics and Gynecology, Tohoku University Graduate School of Medicine, Sendai, 980-8575 Japan; 7 Department of Development and Environmental Medicine, Tohoku University Graduate School of Medicine, Sendai, 980-8575 Japan; 8 Department of Informative Genetics, Environment and Genome Research Center, Tohoku University Graduate School of Medicine, Sendai, 980-8575 Japan; 9 Department of Pediatrics, Tohoku University Hospital, Sendai, 980-8574 Japan; 10 Department of Feto-Maternal Medical Science, Tohoku University Graduate School of Medicine, 2-1, Seiryo-machi, Aoba-ward, Sendai, Miyagi 980-8575 Japan; 11 Nagoya City University, Nagoya, Japan; 12 National Institute for Environmental Studies, Tsukuba, Japan; 13 National Center for Child Health and Development, Tokyo, Japan; 14 Hokkaido University, Sapporo, Japan; 15 Fukushima Medical University, Fukushima, Japan; 16 Chiba University, Chiba, Japan; 17 Yokohama City University, Yokohama, Japan; 18 University of Yamanashi, Chuo, Japan; 19 University of Toyama, Toyama, Japan; 20 Kyoto University, Kyoto, Japan; 21 Osaka University, Suita, Japan; 22 Hyogo Medical University, Nishinomiya, Japan; 23 Tottori University, Yonago, Japan; 24 Kochi University, Nankoku, Japan; 25 Kyushu University, Fukuoka, Japan; 26 Kumamoto University, Kumamoto, Japan

**Keywords:** Gastrointestinal system, Dentistry, Disease prevention, Paediatrics, Patient education, Public health, Gastroenterology, Health care, Health occupations, Risk factors

## Abstract

Functional constipation is the most common gastrointestinal disorder during childhood. Oral stimulation (mastication and toothbrushing) reportedly improves bowel movements, but the association between daily toothbrushing behavior and functional constipation remains unknown. Data (n = 83,660) from fetal records (n = 104,059) in the Japan Environment and Children’s Study, an ongoing prospective birth cohort, were analyzed to investigate the impact of daily toothbrushing frequency on functional constipation during childhood, using ROME III diagnostic criteria. After multiple imputations of data acquired from self-reported questionnaires, a multivariable binomial logistic regression analysis was used with adjustments for several maternal and child-related variables. Functional constipation was observed in 10,123 (12.1%) and 8,820 (10.5%) participants at 3 and 4 years postpartum, respectively. Using the appropriate frequency of daily toothbrushing (twice or more) as a reference, the odds of functional constipation increased with decreasing daily toothbrushing frequency after covariate adjustments involving daily feeding frequency. The adjusted odds ratio (OR) for functional constipation in participants without daily toothbrushing behavior (less than once a day) at 4 years postpartum was 1.87 (95% confidence interval [CI] 1.34–2.61). Similarly, the adjusted OR (95% CI) for chronic functional constipation (at both 3 and 4 years postpartum) in participants without daily toothbrushing behavior (less than once a day) at 2 years postpartum was 1.62 (1.14–2.31). Functional constipation during childhood was associated with decreased daily toothbrushing frequency. Although the underlying mechanism of daily toothbrushing behavior in prompting bowel movements remains unclear, it is a major basis of self-health management in children that plays an important role in managing functional constipation.

## Introduction

Constipation is a common gastrointestinal disorder with an estimated prevalence of approximately 15%^1,2^ in a population-based survey and affects up to 29.6% of children globally^3,4^. Functional constipation (‘normal-transit’ constipation) is the most common form of constipation managed by clinicians^5^ and is characterized by straining or discomfort symptoms, such as difficult or infrequent bowel movements, painful defecation, passage of hard stools, and/or sensation of incomplete evacuation of stool^3,4^. Symptoms related to functional constipation can distress the quality of life of children and their families and increase healthcare expenses due to medical utilization and treatment^2,4^. Oral stimulation, such as mastication with feeding and/or gum chewing, has been clinically applied to facilitate bowel motility and improve defecation in functional constipation^6,7^. Furthermore, an impressive study by Esfandiari et al.^8^, employing a toothbrushing intervention for 5 min twice a day, demonstrated a significant improvement in severe functional constipation in patients with spinal cord injury.

Daily toothbrushing behavior plays a key role in preventing oral infectious diseases, such as dental caries and gingivitis^9,10^, and brushing teeth twice daily using fluoridated toothpaste, particularly during early childhood, is recommended as a highly effective strategy for preventing early childhood dental caries (ECC)^10,11^. Moreover, toothbrushing is one of the major self-health management behaviors. Hakeem et al.^12^ revealed daily toothbrushing frequency as a potential reference for estimating health literacy. Therefore, given that daily toothbrushing habits fundamentally promote an individual’s overall health and well-being^13,14^, evidence using population-based data to examine whether appropriate daily toothbrushing behavior contributes to reducing the risk of functional constipation in childhood would be valuable.

This study utilized a dataset from the Japan Environment and Children’s Study (JECS), an ongoing nationwide multicenter prospective birth cohort study, to investigate the impact of daily toothbrushing frequency on the prevalence of functional constipation during early childhood.

## Results

The baseline characteristics of the participants based on the prevalence of childhood functional constipation at each age are presented in Table 1. Functional constipation was prevalent in 10,123 (12.1%) and 8820 (10.5%) children at 3 and 4 years postpartum, respectively. It should be noted that the Kaup index at each age point is normally distributed with a symmetric histogram.

**Table 1:** Baseline characteristics of participants with and without functional constipation.

Functional constipation	3 years postpartum		4 years postpartum	
	Absence, n (%)	Presence, n (%)	Absence, n (%)	Presence, n (%)
	73,537 (87.9)	10,123 (12.1)	74,840 (89.5)	8820 (10.5)
Maternal age at delivery, Mean (SD)	31.4 (4.5)	31.3 (4.6)	31.4 (4.5)	31.5 (4.6)
Kaup index, Mean (SD)	16.0 (1.3)	16.0 (1.3)	15.7 (1.2)	15.7 (1.2)
Feeding frequency per day at 2 years postpartum, Mean (SD)	3.0 (0.2)	3.0 (0.3)	3.0 (0.2)	3.0 (0.3)
Toothbrushing frequency per day at 2 years postpartum				
Twice or more	35,512 (88.5)	4596 (11.5)	36,265 (90.4)	3843 (9.6)
Once	37,539 (87.4)	5432 (12.6)	38,085 (88.6)	4886 (11.4)
Less than once	486 (83.6)	95 (16.4)	490 (84.4)	91 (15.6)
At 4 years postpartum				
Twice or more	55,921 (88.2)	7497 (11.8)	57,079 (90.0)	6339 (10.0)
Once	17,405 (87.1)	2587 (12.9)	17,560 (87.8)	2432 (12.2)
Less than once	211 (84.3)	39 (15.7)	201 (80.5)	49 (19.5)
Parental-supervised toothbrushing at 2 years postpartum				
Presence	72,290 (87.9)	9921 (12.1)	73,608 (89.5)	8603 (10.5)
Absence	1247 (86.1)	202 (13.9)	1232 (85.0)	217 (15.0)
At 4 years postpartum				
Presence	73,088 (87.9)	10,059 (12.1)	74,395 (89.5)	8752 (10.5)
Absence	449 (87.6)	64 (12.4)	445 (86.7)	68 (13.3)
Child’s sex				
Male	38,122 (89.0)	4732 (11.0)	38,249 (89.3)	4605 (10.7)
Female	35,415 (86.8)	5391 (13.2)	36,591 (89.7)	4215 (10.3)
Maternal parity				
Primiparae	30,468 (86.2)	4858 (13.8)	30,818 (87.2)	4508 (12.8)
Multiparae	43,069 (89.1)	5265 (10.9)	44,022 (91.1)	4312 (8.9)
Household income (million yen/ year)				
< 2	3783 (84.9)	675 (15.1)	3845 (86.3)	613 (13.7)
2 to < 4	24,616 (87.3)	3566 (12.7)	25,070 (89.0)	3112 (11.0)
4 to < 6	25,109 (88.3)	3326 (11.7)	25,534 (89.8)	2901 (10.2)
≥ 6	20,029 (88.7)	2556 (11.3)	20,391 (90.3)	2194 (9.7)
Maternal educational attainment				
High school or less	24,651 (86.7)	3775 (13.3)	25,085 (88.2)	3341 (11.8)
Junior college	31,712 (88.1)	4289 (11.9)	32,274 (89.6)	3727 (10.4)
University or higher	17,174 (89.3)	2059 (10.7)	17,481 (90.9)	1752 (9.1)
Maternal smoking habit				
Never	44,716 (88.7)	5714 (11.3)	45,416 (90.1)	5014 (9.9)
Stopped	26,115 (87.0)	3900 (13.0)	26,709 (89)	3306 (11)
Smoking	2706 (84.2)	509 (15.8)	2715 (84.5)	500 (15.5)
Maternal alcohol intake				
Never	25,736 (88.3)	3402 (11.7)	26,125 (89.7)	3013 (10.3)
Stopped	40,330 (87.5)	5741 (12.5)	41,113 (89.2)	4958 (10.8)
Drinking	7471 (88.4)	980 (11.6)	7602 (90.0)	849 (10.0)
Congenital diseases				
Absence	66,852 (88.0)	9089 (12.0)	68,076 (89.6)	7865 (10.4)
Presence	6685 (86.6)	1034 (13.4)	6764 (87.6)	955 (12.4)

SD = standard deviation.

The crude and adjusted odd ratios (ORs) of daily toothbrushing frequency at 2 and 4 years postpartum (Table 2) for functional constipation were calculated using multivariable logistic regression analysis. The OR (95% confidence interval [CI]) for functional constipation at both 3 and 4 years postpartum in the adjusted model for all covariates tended to increase with a lower frequency in daily toothbrushing at both 2 and 4 years postpartum. In particular, the adjusted ORs (95% CIs) for functional constipation in participants without habitual daily toothbrushing (less than once per day group) at 2 years postpartum were 1.46 (1.12–1.89) and 1.38 (1.05–1.81) at 3 and 4 years postpartum, respectively. Additionally, a similar tendency was observed in the analysis targeting the association of functional constipation with daily toothbrushing frequency at 4 years postpartum; however, the association of lower toothbrushing frequency at 4 years postpartum with functional constipation at 3 years postpartum was weaker in the group with once-daily toothbrushing (1.10 [1.04–1.16]) and not statistically significant in the group without daily habitual toothbrushing (1.30 [0.87–1.95]). When categorizing participants into the target group with lower daily toothbrushing frequency (once per day or less), it is noteworthy that the adjusted model for all covariates demonstrated a significant association with functional constipation at 3 years postpartum (1.10 [1.05–1.16], *p* < 0.001).

**Table 2:** Association of daily toothbrushing frequency at 2 and 4 years postpartum with functional constipation.

Daily toothbrushing frequency at 2 years postpartum	Twice or more	Once	*p*-value	Less than once	*p*-value
Presence of constipation at 3 years of age, n (%)	4596 (11.5)	5432 (12.6)		95 (16.4)	
Crude	Ref	1.12 (1.07–1.17)	< 0.001	1.52 (1.20–1.92)	< 0.001
Model 1^a^		1.11 (1.06–1.16)	< 0.001	1.55 (1.23–1.96)	< 0.001
Model 2^b^		1.12 (1.07–1.17)	< 0.001	1.49 (1.17–1.88)	0.001
Model 3^c^		1.12 (1.07–1.17)	< 0.001	1.46 (1.12–1.89)	0.005
Presence of constipation at 4 years of age, n (%)	3,843 (9.6)	4,886 (11.4)		91 (15.6)	
Crude	Ref	1.21 (1.16–1.27)	< 0.001	1.74 (1.37–2.22)	< 0.001
Model 1^a^		1.18 (1.12–1.23)	< 0.001	1.71 (1.34–2.18)	< 0.001
Model 2^b^		1.18 (1.13–1.24)	< 0.001	1.61 (1.26–2.06)	< 0.001
Model 3^c^		1.18 (1.13–1.24)	< 0.001	1.38 (1.05–1.81)	0.020

Daily toothbrushing frequency at 4 years postpartum	Twice or more	Once	*p*-value	Less than once	*p*-value
Presence of constipation at 3 years of age, n (%)	7497 (11.8)	2587 (12.9)		39 (15.7)	
Crude	Ref	1.11 (1.05–1.17)	< 0.001	1.38 (0.93–2.07)	0.113
Model 1^a^		1.11 (1.05–1.17)	< 0.001	1.42 (0.95–2.12)	0.086
Model 2^b^		1.10 (1.05–1.16)	< 0.001	1.34 (0.89–2.00)	0.157
Model 3^c^		1.10 (1.04–1.16)	< 0.001	1.30 (0.87–1.95)	0.196
Presence of constipation at 4 years of age, n (%)	6339 (10.0)	2432 (12.2)		49 (19.5)	
Crude	Ref	1.25 (1.18–1.31)	< 0.001	2.19 (1.57–3.04)	< 0.001
Model 1^a^		1.22 (1.16–1.29)	< 0.001	2.16 (1.55–3.01)	< 0.001
Model 2^b^		1.21 (1.15–1.28)	< 0.001	1.99 (1.43–2.77)	< 0.001
Model 3^c^		1.21 (1.15–1.27)	< 0.001	1.87 (1.34–2.61)	< 0.001

Odds ratio (95% confidence interval) (all such values).

^a^Adjusted for maternal age, parity status, and child’s sex.

^b^Additionally adjusted for maternal (educational attainment, smoking, and drinking habit), and children’s factors (z-score of Kaup index at each time point, daily feeding frequency, and prevalence of congenital disease[s]), and household income with Model 1.

^c^Additionally adjusted for parental-supervised toothbrushing at each time point with Model 2.

We focused on the association between daily toothbrushing frequency and chronic functional constipation (Table 3). The baseline characteristics associated with the prevalence of chronic functional constipation in the participants are presented in Table S1. The prevalence of chronic functional constipation at both 3 and 4 years postpartum was 3659 (4.4%). Meanwhile, 40,108 (47.9%) and 63,418 (75.8%) participants reported toothbrushing twice daily at 2 and 4 years postpartum, respectively. Using healthy participants as the reference, the crude and adjusted ORs for chronic functional constipation consistently increased with a lower frequency in daily tooth brushing at both 2 and 4 years postpartum. The ORs (95% CIs) for chronic functional constipation in ‘once’ and ‘less than once’ groups of daily toothbrushing frequency at 2 years postpartum were 1.22 (1.14–1.31) and 1.62 (1.14–2.31), respectively; regarding toothbrushing behavior at 4 years postpartum, the ORs were 1.22 (1.13–1.31) and 1.60 (0.90–2.86), respectively. When focusing on lower daily toothbrushing frequency (once per day or less), it is important to note that the adjusted model for all covariates revealed a significant association with chronic functional constipation (1.22 [1.13–1.32], *p* < 0.001). Significant associations between chronic functional constipation and the presence of parent-supervised toothbrushing and daily feeding frequency were not consistently observed in the adjusted model for all covariates (data not shown).

**Table 3:** Association of daily toothbrushing frequency at 2 and 4 years postpartum with chronic functional constipation.

Daily toothbrushing frequency at 2 years postpartum	Twice or more	Once	*p*-value	Less than once	*p*-value
Chronic presence, n (%)	1567 (3.9)	2054 (4.8)		38 (6.6)	
Crude	Ref	1.23 (1.15–1.32)	< 0.001	1.73 (1.22–2.46)	0.002
Model 1^a^		1.21 (1.13–1.30)	< 0.001	1.73 (1.22–2.46)	0.002
Model 2^b^		1.22 (1.14–1.31)	< 0.001	1.64 (1.15–2.34)	0.006
Model 3^c^		1.22 (1.14–1.31)	< 0.001	1.62 (1.14–2.31)	0.008

Daily toothbrushing frequency at 4 years postpartum	Twice or more	Once	*p*-value	Less than once	*p*-value
Chronic presence, n (%)	2626 (4.1)	1015 (5.1)		18 (7.0)	
Crude	Ref	1.24 (1.15–1.34)	< 0.001	1.73 (0.97–3.08)	0.063
Model 1^a^		1.22 (1.13–1.32)	< 0.001	1.75 (0.98–3.12)	0.060
Model 2^b^		1.22 (1.13–1.32)	< 0.001	1.61 (0.91–2.88)	0.104
Model 3^c^		1.22 (1.13–1.31)	< 0.001	1.60 (0.90–2.86)	0.109

Odds ratio (95% confidence interval) (all such values).

^a^Adjusted for maternal age, parity status and infant’s sex.

^b^Additionally adjusted for maternal (educational attainment, smoking and drinking habit), and children’s factors (z-score of Kaup index at 4 years postpartum, daily feeding frequency, and prevalence of congenital disease[s]), and household income with Model 1.

^c^Additionally adjusted for parental-supervised toothbrushing at each time point with Model 2.

## Discussion

The key findings of this study, using nationwide data from a large-scale birth cohort study in Japan, showed that a decreased frequency of daily toothbrushing behavior prospectively increased the risk of functional constipation during childhood. Although it certainly promotes children’s oral health, a better understanding of the appropriate association between daily toothbrushing and bowel habits would provide valuable information for planning and supporting health and behavioral development in children.

Daily toothbrushing behavior is a major basis for self-health management^12,14^. Similar to the study by Esfandiari et al.^8^, in which chronic constipation distinctly improved after intervention with toothbrushing twice daily for 5 min after feeding in patients with spinal cord injury, our findings also indicated a favorable association of daily frequency of toothbrushing habits with a reduced risk of functional constipation in children; however, the potential causality remains unclear. Interestingly, in our results, the prevalence of functional constipation at 3 years postpartum was significantly associated with a lower daily toothbrushing frequency at 2 years, but not at 4 years postpartum. Since the acquisition of toothbrushing behavior in early childhood starts with tooth eruption and tends to become habitual with growth^15,16^, the proportion of children with twice-daily toothbrushing increased from half at 2 years postpartum (47.9%) to three-fourths at 4 years postpartum (75.8%), as shown in Table 1. Thus, considering the prospective impact of appropriate daily toothbrushing on the prevalence of functional constipation, acquiring appropriate toothbrushing behavior in early childhood would contribute to individual health promotion, including bowel habits.

There is a lack of studies focusing on the underlying mechanism of daily toothbrushing behavior on prompting bowel movements. However, several studies involving several oral stimulations such as feeding, mastication, or taste in human and animal models induced bowel motility and gut secretion through the cephalic-vagal reflex via the autonomic nerve and humoral systems^17,18^. Additionally, based on the robust correlation between oral and bowel microbiota^19,20^, daily toothbrushing, the most effective means of maintaining oral hygiene orchestrated by oral bacteria, might indirectly provide beneficial effects on gut microbial composition, playing vital roles in the bowel environment^21,22^. Meanwhile, although there is fundamentally higher interindividual variability in bowel habits, reinforcement of both appropriate daily toothbrushing and bowel habits in children depends on parental childcare, health literacy, and their behaviors^23–25^. Therefore, the influence of potential confounding factors associated with daily parenting quality in terms of toothbrushing and bowel training cannot be ruled out. Since bowel habits contribute to healthy child development in both physical and mental aspects, appropriate daily toothbrushing habits play a key role not only in preventing ECC^11^ but also in contributing to the extensive health benefits for children.

This study had several strengths and limitations. One of the strengths is that the JECS dataset utilized in our study was obtained from the Japanese nationwide survey that included almost half of all infants born in multiple study regions from 2011 to 2014^26–28^. Thus, the findings, mostly based on the general Japanese population, allowed us to examine the behavioral onset of daily toothbrushing in children and its association with children’s functional constipation in comparison with abundant healthy participants. Meanwhile, as a main dataset limitation, this study’s data collection methods did not include queries about clinical histories and self-management of functional constipation. Considering that medications for constipation in children can be ruled out because they generally start around preschool age, home remedies used to relieve chronic symptoms are necessary to verify their association with parental socioeconomic and behavioral determinants. In particular, the benefits of daily intake of dietary fiber and probiotics in improving constipation are well known. Thus, in addition to the daily feeding frequency included in the analysis, further studies that include more information on dietary habits are warranted.

In conclusion, a decreased frequency of daily toothbrushing was prospectively associated with the prevalence of functional constipation in children. As a major basis of self-health management in children, daily toothbrushing behavior plays an important role in contributing to the child’s overall health and development.

## Methods

### Study design and participants

The JECS conformed to the ethical guidelines of the 1975 Declaration of Helsinki (revised in 2008). The JECS protocol was reviewed and approved by the Ministry of the Environment’s Institutional Review Board on Epidemiological Studies and the Ethics Committees of all the participating institutions (No. 100910001), the details of which have been published^26,29^. The aim and procedure of the study were explained to all participants, and written informed consent was obtained prior to their participation. The early childhood data were collected at 2, 3, and 4 years postpartum using follow-up questionnaires. Based on the lower withdrawal rate (approximately 5%), the JECS consistently achieves a response rate of over 80%. Specifically, the response rates were 87.3%, 84.2%, and 80.5% at 2, 3, and 4 years postpartum, respectively. This study was conducted as a part of the JECS, and anonymized data were utilized. This study relied on the jecs-ta-20190930-qsn and jecs-qa-20210401 datasets released in October 2019 and April 2021, respectively.

**Fig. 1:**
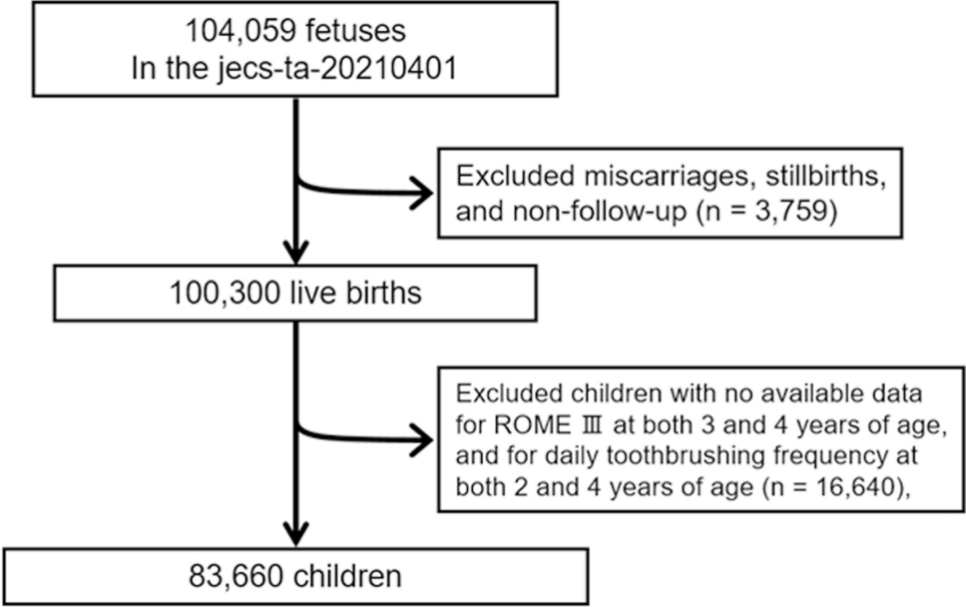
Flow chart of the study participants.

In the JECS, pregnant women were recruited during their first prenatal examination from cooperating healthcare providers and local government offices between January 2011 and March 2014. After the participating mothers completed the self-administered questionnaire, medical doctors and trained nurses performed clinical measurements and summarized the medical record transcripts. Of the 104,059 fetuses enrolled from 15 regional centers in the JECS, 3759 comprised miscarriages and stillbirths or were untraceable, and 16,640 participants’ mothers did not respond to the questionnaires regarding the ROME III diagnostic criteria in children at both 3 and 4 years postpartum, or daily toothbrushing frequency at both 2 and 4 years postpartum. After excluding these fetuses and children from the analysis, the final sample comprised 83,660 children (Fig. 1).

### Functional constipation (outcome measure)

The prevalence of functional constipation in participants was determined using the ROME III diagnostic criteria, which is a standard set for screening childhood functional gastrointestinal disorders^4,30^. The following items associated with functional constipation lasting for a minimum of 1 month were evaluated: (1) two or fewer defecations per week, (2) at least one episode of fecal incontinence per week, (3) a history of excessive stool retention, (4) history of painful or hard bowel movements, (5) presence of a large fecal mass in the rectum, and (6) history of large-diameter stools that may obstruct the toilet^4,30^. Parents or caregivers of the participants were enquired regarding the above questions via mailed questionnaires in Japanese version at 3 and 4 years postpartum. Participants with two or more positive items on the questionnaire were defined as having childhood functional constipation at each time point. Additionally, considering the established consistent symptoms of functional constipation, participants with functional constipation at both time points (presence at both 3 and 4 years postpartum) were classified into the chronic functional constipation group and included for analyses as a binary variable: the absence and presence groups of chronic functional constipation.

### Daily toothbrushing frequency (main exposure measure)

Daily toothbrushing frequency in children at 2 and 4 years postpartum was assessed using the following question: ‘How many times do you/does your child brush his or her teeth every day?’ in a questionnaire sent to the participants’ mothers^25,31^. The response options were: never, once per day, twice per day, three times per day, and four or more times per day. As internationally recommended in the pediatric dental guidelines, toothbrushing twice a day is the most effective strategy in preventing ECC^11,32^. The participants in the present study were categorized into three groups: the reference group (≥ 2 times per day), low-frequency group (once per day), and very-low-frequency group (less than once per day), or into two groups: the reference (≥ 2 times per day) and lower-frequency (once per day or less) groups.

### Covariates

The detailed design of the questionnaire has been previously described^25–27^. Briefly, sociodemographic characteristics, lifestyle, and health status variables in mother-infant dyads, which reportedly confound results in health and health-related behaviors^33,34^, were included as covariates in the analysis models. Information, such as annual household income, maternal educational level, smoking habits, and drinking intake, were assessed using a self-administered questionnaire provided to the mothers during pregnancy. Data on maternal age at delivery, parity status, and sex of the child were retrieved from the medical record transcripts. All data were retrieved using medical record transcripts and self-administered questionnaires. Additionally, the prevalence of congenital anomalies was ascertained in 7719 (9.2%) infants from the medical record transcripts and questionnaires. Details regarding the data processing, validation, and verification of congenital anomalies have been previously described^29,35^.

From the data, the participants were categorized into different groups based on the following variables: annual household income in Japanese yen (< 2 million, 2–4 million, 4–6 million, or ≥ 6 million); maternal educational level (junior or high school, junior college [technical or junior college], or university [university or graduate]); maternal smoking history (never, stopped smoking before or during pregnancy [previously did, but quit before realizing current pregnancy; previously did, but quit after realizing current pregnancy], currently smoking); alcohol intake (never, stopped drinking, or current drinker); sex of the child (male or female); parity status (primiparae or multiparae); and prevalence of congenital disease (presence or absence). The degree of obesity in participants according to age was estimated using the Kaup index (g/cm^2^ × 10, similar to body mass index), which has been applied to children aged between 2 and 5 years. Additionally, the Kaup index was transformed into z-scores using the standard z-score calculation formula and incorporated as a covariate in the analysis. In addition to the Kaup index z-scores, maternal age at delivery and daily feeding frequency at 2 years postpartum were included as continuous variables in the analysis.

### Statistical analysis

Of the 83,660 participants, the missing data on functional constipation at 3 and 4 years postpartum was 7894 (9.4%) and 6813 (8.1%) children, respectively. Regarding daily toothbrushing frequency, 2274 (2.7%) and 5617 (6.7%) participants had missing data at 2 and 4 years postpartum, respectively. In order to apply the missing data, multiple imputations using the “missing at random” (MAR) assumption were performed using the multivariate normal imputation method^36^. An imputation model that included all the variables (including the main exposure and outcome variables) used in the main analysis was independently applied to 10 copies of the data, each of which contained suitably imputed missing values. According to Rubin’s rules, the imputed values of variables should be estimated using the means and adjusted standard errors yielded by the observed data^37^. The missing values according to variables are summarized in Table S2.

As summarized in Table 1, the maternal age at delivery, Kaup indices, and daily feeding frequencies are presented as mean and standard deviation. Categorical variables are presented as numbers and percentiles. To calculate the ORs and 95% CIs for functional constipation, using the healthy control group as a reference, binomial logistic regression analysis, including potential covariates using the simultaneous method, was performed. For crude or adjusted analyses using the aforementioned covariates, Model 1 was analyzed after adjusting for maternal age at delivery, parity status, and the child’s sex. Model 2 also included maternal factors (educational attainment, smoking, and drinking habits), child-related factors (Kaup index, daily feeding frequency, and prevalence of congenital diseases), and household income, in addition to the variables included in Model 1. Moreover, Model 3 included the presence of parent-supervised toothbrushing at each age (2 and 4 years postpartum) as a categorical variable (Tables 2 and 3). Focusing on chronic functional constipation associated with daily tooth brushing frequency in children, a secondary analysis of participants with chronic functional constipation was also performed. All statistical analyses were performed using IBM SPSS Statistics software (version 24.0; IBM Corp., Armonk, NY, USA). Statistical significance was set at *p*-value < 0.05.

## Supplementary Information

Supplementary Information 1.

Supplementary Information 2.

Supplementary Information 3.

## Data Availability

Data are unsuitable for public deposition due to ethical restrictions and legal framework of Japan. It is prohibited by the Act on the Protection of Personal Information (Act No. 57 of 30 May 2003, amendment on 9 September 2015) to publicly deposit the data containing personal information. Ethical Guidelines for Medical and Health Research Involving Human Subjects enforced by the Japan Ministry of Education, Culture, Sports, Science and Technology and the Ministry of Health, Labour and Welfare also restricts the open sharing of the epidemiologic data. All inquiries about access to data should be sent to: jecs-en@nies.go.jp. The person responsible for handling enquiries sent to this e-mail address is Dr Shoji F. Nakayama, JECS Programme Office, National Institute for Environmental Studies.

## References

[1] Higgins, P. D. & Johanson, J. F. Epidemiology of constipation in North America: a systematic review. *Am. J. Gastroenterol.* **99**, 750–759 (2004). 15089911 10.1111/j.1572-0241.2004.04114.x

[2] Bharucha, A. E. & Lacy, B. E. Mechanisms, evaluation, and management of chronic constipation. *Gastroenterology* **158**, 1232–1249 (2020). 31945360 10.1053/j.gastro.2019.12.034 PMC7573977

[3] Mugie, S. M., Benninga, M. A. & Di Lorenzo, C. Epidemiology of constipation in children and adults: a systematic review. *Best Pract. Res. Clin. Gastroenterol.* **25**, 3–18 (2011). 21382575 10.1016/j.bpg.2010.12.010

[4] Lee, Y. J. & Park, K. S. Understanding the changes in diagnostic criteria for functional constipation in pediatric patients: From Rome III to Rome IV. *J. Neurogastroenterol. Motil.* **25**, 3–5 (2019). 30646474 10.5056/jnm18199 PMC6326195

[5] Andrews, C. N. & Storr, M. The pathophysiology of chronic constipation. *Can. J. Gastroenterol.* **25**(Suppl B), 16B-21B (2011). 22114753 PMC3206564

[6] Friedman, G. Diet and the irritable bowel syndrome. *Gastroenterol. Clin. N. Am.* **20**, 313–324 (1991). 2066155

[7] Yenigul, N. N., Aydogan Mathyk, B., Aslan Cetin, B., Yazici Yilmaz, F. & Ayhan, I. Efficacy of chewing gum for improving bowel function after cesarean sections: a randomized controlled trial. *J. Matern. Fetal Neonatal Med.* **33**, 1840–1845 (2020). 30606082 10.1080/14767058.2018.1531122

[8] Esfandiari, E. et al. Novel effects of traditional wooden toothbrush on bowel motility symptoms in spinal cord injury patients; findings from a pilot quasi-experimental study. *Int. J. Prev. Med.* **8**, 46 (2017). 28706615 10.4103/ijpvm.IJPVM_174_16 PMC5499390

[9] Marinho, V. C., Higgins, J. P., Sheiham, A. & Logan, S. Fluoride toothpastes for preventing dental caries in children and adolescents. *Cochrane Database Syst. Rev.* **1**, CD002278 (2003). 10.1002/14651858.CD002278 PMC8439270 12535435

[10] American Academy of Pediatrics. Policy on early childhood caries (ECC): Classifications, consequences, and preventive strategies. *Pediatr. Dent.* **39**, 59–61 (2017). 29179321

[11] IAPD Bangkok Declaration. Early childhood caries. *Int. J. Paediatr. Dent.* **29**, 384–386 (2019). 31099129 10.1111/ipd.12490

[12] Hakeem, F. F. et al. The association between electronic health literacy and oral health outcomes among dental patients in Saudi Arabia: A cross-sectional study. *Healthcare* **11**(12), 1804. 10.3390/healthcare11121804 (2023). 37372921 10.3390/healthcare11121804 PMC10298494

[13] Tsuchiya, M. et al. Excessive game playing is associated with poor toothbrushing behavior among athletic children: A cross-sectional study in Miyagi, Japan. *Tohoku J. Exp. Med.* **241**, 131–138 (2017). 28190825 10.1620/tjem.241.131

[14] Bernabe, E. et al. The influence of sense of coherence on the relationship between childhood socioeconomic status and adult oral health-related behaviours. *Community Dent. Oral Epidemiol.* **37**, 357–365 (2009). 19614720 10.1111/j.1600-0528.2009.00483.x

[15] Dickson-Swift, V. et al. Supervised toothbrushing programs in primary schools and early childhood settings: A scoping review. *Community Dent. Health* **34**, 208–225 (2017). 29119741 10.1922/CDH_4057Dickson-Swift18

[16] Astrom, A. N. Stability of oral health-related behaviour in a Norwegian cohort between the ages of 15 and 23 years. *Community Dent. Oral Epidemiol.* **32**, 354–362 (2004). 15341620 10.1111/j.1600-0528.2004.00174.x

[17] Noble, E. J., Harris, R., Hosie, K. B., Thomas, S. & Lewis, S. J. Gum chewing reduces postoperative ileus? A systematic review and meta-analysis. *Int. J. Surg.* **7**, 100–105 (2009). 19261555 10.1016/j.ijsu.2009.01.006

[18] Yaoita, F. et al. Impact of habitual chewing on gut motility via microbiota transition. *Sci. Rep.* **12**, 13819 (2022). 35970869 10.1038/s41598-022-18095-x PMC9378666

[19] Fourie, N. H. et al. The microbiome of the oral mucosa in irritable bowel syndrome. *Gut Microbes* **7**, 286–301 (2016). 26963804 10.1080/19490976.2016.1162363 PMC4988452

[20] Docktor, M. J. et al. Alterations in diversity of the oral microbiome in pediatric inflammatory bowel disease. *Inflamm Bowel Dis.* **18**, 935–942 (2012). 21987382 10.1002/ibd.21874 PMC4208308

[21] Kobayashi, R., Ogawa, Y., Hashizume-Takizawa, T. & Kurita-Ochiai, T. Oral bacteria affect the gut microbiome and intestinal immunity. *Pathog. Dis.* **78**, ftaa024 (2020). 32504490 10.1093/femspd/ftaa024

[22] Olsen, I. & Yamazaki, K. Can oral bacteria affect the microbiome of the gut?. *J. Oral. Microbiol.* **11**, 1586422 (2019). 30911359 10.1080/20002297.2019.1586422 PMC6427756

[23] Reeves, P. T. et al. Development and assessment of a pictographic pediatric constipation action plan. *J. Pediatr.* **229**, 118–126 (2021). 33068567 10.1016/j.jpeds.2020.10.001 PMC7557278

[24] Trubey, R. J., Moore, S. C. & Chestnutt, I. G. Children’s toothbrushing frequency: the influence of parents’ rationale for brushing, habits and family routines. *Caries Res.* **49**, 157–164 (2015). 25634461 10.1159/000365152

[25] Tsuchiya, S. et al. Influence of maternal postpartum depression on children’s toothbrushing frequency. *Community Dent. Oral Epidemiol.* **50**, 300–310 (2022). 34117651 10.1111/cdoe.12672

[26] Kawamoto, T. et al. Rationale and study design of the Japan environment and children’s study (JECS). *BMC Public Health* **14**, 25 (2014). 24410977 10.1186/1471-2458-14-25 PMC3893509

[27] Michikawa, T. et al. Baseline profile of participants in the Japan Environment and Children’s Study (JECS). *J. Epidemiol.* **28**, 99–104 (2018). 29093304 10.2188/jea.JE20170018 PMC5792233

[28] Tsuchiya, M. et al. Prospective association of short sleep duration in newborns with bruxism behavior in children: The Japan Environment and Children’s Study (JECS). *Sleep Med.* **100**, 71–78 (2022). 36029753 10.1016/j.sleep.2022.07.018

[29] Mezawa, H. et al. Prevalence of congenital anomalies in the Japan Environment and Children’s Study. *J. Epidemiol.* **29**, 247–256 (2019). 30249945 10.2188/jea.JE20180014 PMC6556438

[30] Motoki, N. et al. Impact of breastfeeding during infancy on functional constipation at 3 years of age: The Japan Environment and Children’s Study. *Int. Breastfeed J.* **18**, 57 (2023). 37926840 10.1186/s13006-023-00592-y PMC10626743

[31] Tsuchiya, S. et al. Prospective association between maternal bonding disorders and child toothbrushing frequency: A cross-sectional study of the Japan Environment and Children’s Study. *Int. J. Paediatr. Dent.* **32**, 56–65 (2022). 33764606 10.1111/ipd.12791

[32] American Academy of Pediatric Dentistry. Policy on early childhood caries (ECC): classifications, consequences, and preventive strategies. *Pediatr. Dent.* **39**, 59–61 (2017). 29179321

[33] Knappe, S., Pfarr, A. L., Petzoldt, J., Hartling, S. & Martini, J. Parental cognitions about sleep problems in infants: A systematic review. *Front. Psychiatry* **11**, 554221 (2020). 33408648 10.3389/fpsyt.2020.554221 PMC7779594

[34] Lavigne, G. J., Khoury, S., Abe, S., Yamaguchi, T. & Raphael, K. Bruxism physiology and pathology: an overview for clinicians. *J. Oral Rehabil.* **35**, 476–494 (2008). 18557915 10.1111/j.1365-2842.2008.01881.x

[35] Tsuchiya, S. et al. Association of cleft lip and palate on mother-to-infant bonding: a cross-sectional study in the Japan Environment and Children’s Study (JECS). *BMC Pediat.* **19**, 505 (2019). 10.1186/s12887-019-1877-9 PMC6923825 31862001

[36] Cummings, P. Missing data and multiple imputation. *JAMA Pediatr.* **167**, 656–661 (2013). 23699969 10.1001/jamapediatrics.2013.1329

[37] Rubin, D. B. & Schenker, N. Multiple imputation in health-care databases: an overview and some applications. *Stat. Med.* **10**, 585–598 (1991). 2057657 10.1002/sim.4780100410

