# Supplementary material for: Prospective association of daily toothbrushing frequency and the prevalence of childhood functional constipation: the Japan Environment and Children’s Study
Source: Sci Rep. 2025 Mar 5;15:7753. doi: 10.1038/s41598-025-88562-8 (PMC11882968; doi:10.1038/s41598-025-88562-8)
Supplement: Supplementary file 1 — Supplementary Information 1. [file 41598_2025_88562_MOESM1_ESM.docx]

**Appendix**

Members of the JECS Group as of 2023: Michihiro Kamijima (principal investigator, Nagoya City University, Nagoya, Japan), Shin Yamazaki (National Institute for Environmental Studies, Tsukuba, Japan), Yukihiro Ohya (National Center for Child Health and Development, Tokyo, Japan), Reiko Kishi (Hokkaido University, Sapporo, Japan), Nobuo Yaegashi (Tohoku University, Sendai, Japan), Koichi Hashimoto (Fukushima Medical University, Fukushima, Japan), Chisato Mori (Chiba University, Chiba, Japan), Shuichi Ito (Yokohama City University, Yokohama, Japan), Zentaro Yamagata (University of Yamanashi, Chuo, Japan), Hidekuni Inadera (University of Toyama, Toyama, Japan), Takeo Nakayama (Kyoto University, Kyoto, Japan), Tomotaka Sobue (Osaka University, Suita, Japan), Masayuki Shima (Hyogo Medical University, Nishinomiya, Japan), Seiji Kageyama (Tottori University, Yonago, Japan), Narufumi Suganuma (Kochi University, Nankoku, Japan), Shoichi Ohga (Kyushu University, Fukuoka, Japan), and Takahiko Katoh (Kumamoto University, Kumamoto, Japan).
