# Supplementary material for: Prospective association of daily toothbrushing frequency and the prevalence of childhood functional constipation: the Japan Environment and Children’s Study
Source: Sci Rep. 2025 Mar 5;15:7753. doi: 10.1038/s41598-025-88562-8 (PMC11882968; doi:10.1038/s41598-025-88562-8)
Supplement: Supplementary file 2 — Supplementary Information 2. [file 41598_2025_88562_MOESM2_ESM.docx]

| **Table S1. Baseline characteristics of 83,660 children participating in the JECS (2011-2015)** | | | | | | |
| --- | --- | --- | --- | --- | --- | --- |
|  | ***Chronic functional constipation*** | | | |  |  |
|  | Absence, n (%) | | Presence, n (%) | | |  |
|  | 80,001 (95.6) | | 3,659 (4.4) | | |  |
| ***Maternal age at delivery, Mean (SD)*** | | | |  |  |  |
|  | 31.4 (4.9) | | 31.4 (5.1) | | |  |
| ***Kaup index at 4 years of age, Mean (SD)*** | |  |  |  |  |  |
|  | 15.7 (1.2) | | 15.7 (1.2) | | |  |
| ***Feeding frequency per day at 2 years of age, Mean (SD)*** | | | |  |  |  |
|  | 3.0 (0.2) | | 3.0 (0.3) | | |  |
| ***Toothbrushing frequency per day at 2 years of age*** | | | |  |  |  |
| Twice or more | 38,541 (96.1) | | 1,567 (3.9) | | |  |
| Once | 40,917 (95.2) | | 2,054 (4.8) | | |  |
| Less than once | 543 (93.4) | | 38 (6.6) | | |  |
| ***at 4 years of age*** | | | |  |  |  |
| Twice or more | 60,792 (95.9) | | 2,626 (4.1) | | |  |
| Once | 18,977 (94.9) | | 1,015 (5.1) | | |  |
| Less than once | 232 (93.0) | | 18 (7.0) | | |  |
| ***Parental-supervised toothbrushing at 2 years of age*** | | | |  |  |  |
| Absence | 78,630 (95.6) | | 3,581 (4.4) | | |  |
| Presence | 1,371 (94.6) | | 78 (5.4) | | |  |
| ***at 4 years of age*** | | | |  |  |  |
| Absence | 79,516 (95.6) | | 3,631 (4.4) | | |  |
| Presence | 485 (94.6) | | 28 (5.4) | | |  |
| ***Child's sex*** | | | |  |  |  |
| Male | 41,076 (95.9) | | 1,778 (4.1) | | |  |
| Female | 38,925 (95.4) | | 1,881 (4.6) | | |  |
| ***Maternal parity*** | | | |  |  |  |
| Primiparae | 33,460 (94.7) | | 1,866 (5.3) | | |  |
| Multiparae | 46,541 (96.3) | | 1,793 (3.7) | | |  |
| ***Household income (million yen/ year)*** | | | |  |  |  |
| <2 | 4,202 (94.3) | | 256 (5.7) | | |  |
| 2 to <4 | 26,872 (95.4) | | 1,310 (4.6) | | |  |
| 4 to <6 | 27,258 (95.9) | | 1,177 (4.1) | | |  |
| ≥6 | 21,669 (95.9) | | 916 (4.1) | | |  |
| ***Educational attainment*** | | | |  |  |  |
| High school or less | 27,047 (95.1) | | 1,379 (4.9) | | |  |
| Junior college | 34,431 (95.6) | | 1,570 (4.4) | | |  |
| University or higher | 18,523 (96.3) | | 710 (3.7) | | |  |
| ***Smoking habit*** | | | |  |  |  |
| Never | 48,366 (95.9) | | 2,064 (4.1) | | |  |
| Stopped | 28,617 (95.3) | | 1,398 (4.7) | | |  |
| Smoking | 3,018 (93.9) | | 197 (6.1) | | |  |
| ***Alcohol intake*** | | | |  |  |  |
| Never | 27,885 (95.7) | | 1,253 (4.3) | | |  |
| Stopped | 44,001 (95.5) | | 2,070 (4.5) | | |  |
| Drinking | 8,115 (96.0) | | 336 (4,0) | | |  |
| ***Congenital diseases*** | | | |  |  |  |
| Absence | 72,700 (95.7) | | 3,241 (4.3) | | |  |
| Presence | 7,301 (94.6) | | 418 (5.4) | | |  |
| SD = standard deviation. | |  |  |  |  |  |
