# Supplementary material for: Prospective association of daily toothbrushing frequency and the prevalence of childhood functional constipation: the Japan Environment and Children’s Study
Source: Sci Rep. 2025 Mar 5;15:7753. doi: 10.1038/s41598-025-88562-8 (PMC11882968; doi:10.1038/s41598-025-88562-8)
Supplement: Supplementary file 3 — Supplementary Information 3. [file 41598_2025_88562_MOESM3_ESM.docx]

| **Table S2. Baseline characteristics of missing data (n [%])** | |  |
| --- | --- | --- |
|  | |  |
| ***ROME*** ***III at 3 years postpartum^a^*** | 7,894 (9.4) | |
| ***at 4 years postpartum^a^*** | 6,813 (8.1) | |
| ***Maternal age at delivery*** | 1 (0.0) | |
| ***Kaup index at 3 years postpartum^b^*** | 8,898 (10.6) | |
| ***at 4 years postpartum^b^*** | 7,946 (9.5) | |
| ***Feeding frequency per day at 2 years postpartum*** | 7,346 (8.8) | |
| ***Toothbrushing frequency per day at 2 years postpartum*** | 2,274 (2.7) | |
| ***at 4 years postpartum*** | 5,617 (6.7) | |
| ***Parental-supervised toothbrushing at 2 years postpartum*** | 2,316 (2.8) | |
| ***at 4 years postpartum*** | 6,459 (7.7) | |
| ***Child’s sex*** | 0 (0.0) | |
| ***Maternal parity*** | 2,027 (2.4) | |
| ***Household income (million yen/ year)*** | 5,952 (7.1) | |
| ***Maternal educational attainment*** | 970 (1.2) | |
| ***Maternal smoking habit*** | 289 (0.3) | |
| ***Maternal alcohol intake*** | 188 (0.2) | |
| ***Congenital diseases*** | 0 (0.0) | |

^a^ Participants with at least one missing data in the six items of the ROME III.

^b^ Participants with missing values in either body height or weight.
